# Integrative Binding Sites within Intracellular Termini of TRPV1 Receptor

**DOI:** 10.1371/journal.pone.0048437

**Published:** 2012-10-31

**Authors:** Lenka Grycova, Blanka Holendova, Ladislav Bumba, Jan Bily, Michaela Jirku, Zdenek Lansky, Jan Teisinger

**Affiliations:** 1 Institute of Physiology, v.v.i., Academy of Sciences of the Czech Republic, Prague, Czech Republic; 2 Institute of Microbiology, v.v.i., Academy of Sciences of the Czech Republic, Prague, Czech Republic; Indiana University School of Medicine, United States of America

## Abstract

TRPV1 is a nonselective cation channel that integrates wide range of painful stimuli. It has been shown that its activity could be modulated by intracellular ligands PIP2 or calmodulin (CaM). The detailed localization and description of PIP2 interaction sites remain unclear. Here, we used synthesized peptides and purified fusion proteins of intracellular regions of TRPV1 expressed in *E.coli* in combination with fluorescence anisotropy and surface plasmon resonance measurements to characterize the PIP2 binding to TRPV1. We characterized one PIP2 binding site in TRPV1 N-terminal region, residues F189-V221, and two independent PIP2 binding sites in C–terminus: residues K688-K718 and L777-S820. Moreover we show that two regions, namely F189-V221 and L777-S820, overlap with previously localized CaM binding sites. For all the interactions the equilibrium dissociation constants were estimated. As the structural data regarding C-terminus of TRPV1 are lacking, restraint-based molecular modeling combined with ligand docking was performed providing us with structural insight to the TRPV1/PIP2 binding. Our experimental results are in excellent agreement with our *in silico* predictions.

## Introduction

The vanilloid receptor (TRPV1) is one of the best characterized members of the TRPV subfamily. This nonselective cation channel serves as a polymodal receptor for various potentially harmful signals. Activation is caused by diverse stimuli, such as noxious heat (>43°C), low pH (<5.4) and chemicals such as capsaicin, its analogs and a wide range of other agonists (f. e. resiniferatoxin, anandamide [1], [2], [3], [4], [5]). It is assumed that the TRPV1 channel has six transmembrane domains with a pore domain between the fifth and the sixth segment and as has been recently confirmed by electron microscopy, forms a tetrameric structure with a central localized pore [6]. Both its C- and N- termini are located intracellularly and have been shown to be involved in the regulation of the channel activity [4], [7].

A number of studies have demonstrated that the cytoplasmic regions of TRP channels bind agonists and regulatory molecules such as ATP, calmodulin (CaM) and phosphatidyl inositol-4, 5-bisphosphate (PIP2) [8], [9], [10], [11], [12], [13], [14], [15]. PIP2 is a minor component of the plasma membrane with multiple functions. It is involved in the regulation of many proteins and itself anchors proteins to the plasma membrane through pleckstrin homology (PH) and other domains with known structure [16], [17], [18], [19]. One of its important roles is acting as a source of secondary messengers [20]. As has been reported previously PIP2 regulates the activity of many ion channels including a number of mammalian TRP channels [21]. CaM/PIP2 binding sites have been reported on the TRPC6 C-terminus (CT), and the regulation of CaM binding to the TRPV1 - CT by phosphoinositide has been suggested [12]. However the exact role of PIP2 as a TRPV1 activity modulator remains elusive. Whether PIP2 acts directly on TRPV1 [22] or intervenes via the accessory membrane protein PIRT [23] has been discussed. Moreover the exact molecular mechanism of PIP2-dependent regulation of TRPV1 is still unclear, as well as whether PIP2 works as an activator [24], [25], inhibitor [9] or causes bidirectional modulation [26], [27]. To date several possible PIP2-interacting regions have been proposed within the cytosolic termini of the TRPV1 channel [9], [10], [22].

We show that there are three different regions on the cytoplasmic domains of TRPV1 interacting with PIP2. We found the precise location of the binding sites within the C- and N- termini and we estimated the corresponding binding affinities. Using the combination of biophysical and bioinformatical methods we identified the key residues involved in PIP2 binding in the proximal and distal regions of the C-tail of TRPV1. We showed that regions on C- terminus L777-S820 and N- terminus F189-V221overlap with the CaM binding sites and the third PIP2 binding site K688-K718 occupies the TRP domain on C- terminus, a highly conserved sequence among the members of the TRP ion channel family. We found that the presence of PIP2 prevents the interaction of the TRPV1–CT distal region with CaM, which could play an important role in the regulation of TRPV1.

## Results

### PIP2 Binds to the TRPV1 C-tail Distal Region (712–838)

**Figure 1:**
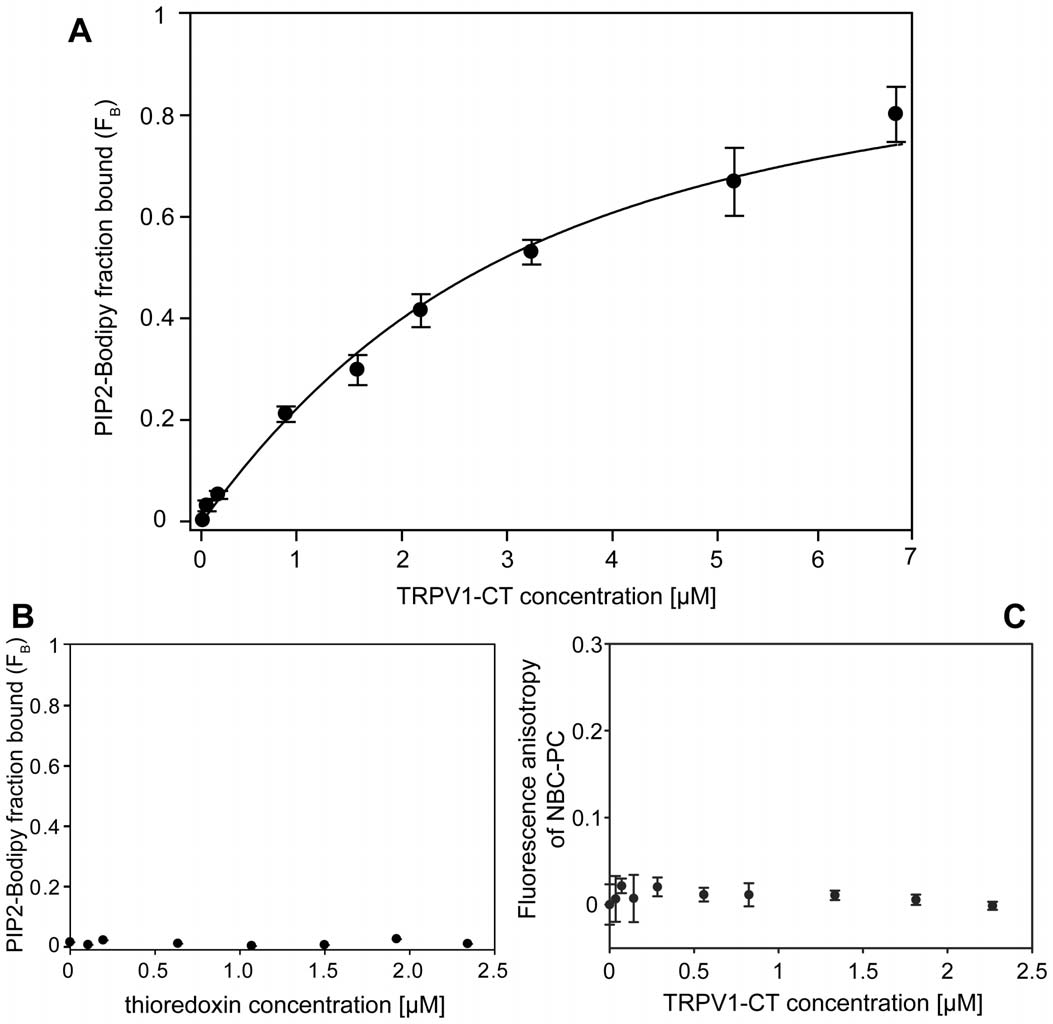
PIP2 binds to the C-terminal distal region of TRPV1. A. Fluorescence anisotropy measurements of interaction between fluorescently labeled phosphatidyl inositol-4, 5-bisphosphate (PIP2-Bodipy) and the distal region of TRPV1 (amino acids 712–838) fusion protein. PIP2-Bodipy (10 nM) was titrated with TRPV1-CT fusion protein WT and the bound fraction (FB) was calculated according to Equation 1 as described in Material and Methods. Binding isotherm and the equilibrium dissociation constant KD (3.48+/−0.93 µM) was determined by fitting the data to the Equation 2 as described in Material and Methods. **B.** Fluorescence anisotropy measurements of interaction between PIP2-Bodipy and thioredoxin. PIP2-Bodipy (10 nM) was titrated with thioredoxin and the bound fraction (FB) of PIP2 Bodipy was calculated as above. **C.** Steady-state fluorescence anisotropy measurement of interaction between fluorescently labeled phosphatidyl choline (NBD–PC) and TRPV1-CT. NBD-PC (10 nM) was titrated with indicated concentrations of TRPV1-CT fluorescence anisotropy was recorded. Values are expressed as the mean ± standard deviation (SD) measured from at least from six independent experiments.

In this report we studied part of the sequence on the C-tail of TRPV1 (amino acids 712–838; henceforth denoted as TRPV1-CT) containing the distal putative PIP2 interaction region. [9] The region was expressed as a fusion protein in E. coli with thioredoxin at the N-terminus and a 6× His tag located at both termini. Proteins (wild type construct and its site directed mutants) were purified using a two-step purification protocol. All expressed proteins were soluble, and expression yields were sufficient to perform spectroscopic and biochemical studies. We were unable to remove the thioredoxin, as the TRPV1-CT alone has a strong tendency to aggregate. The binding of PIP2 to the TRPV1-CT wild-type was investigated using a steady-state fluorescence anisotropy binding assay. A fluorescent PIP2 analogue, PIP2-Bodipy, was titrated with an increasing amount of TRPV1-CT and the steady state anisotropy was measured (Fig. 1A). We observed the binding of PIP2-Bodipy to TRPV1-CT in a 1∶1 ratio and we estimated the equilibrium dissociation constant of the complex formation to be 3.48+/−0.93 µM. In a control experiment, an increasing amount of thioredoxin was titrated to the PIP2 analogue, PIP2-Bodipy. We observed no thioredoxin–PIP2 binding (Fig. 1B).

**Figure 2:**
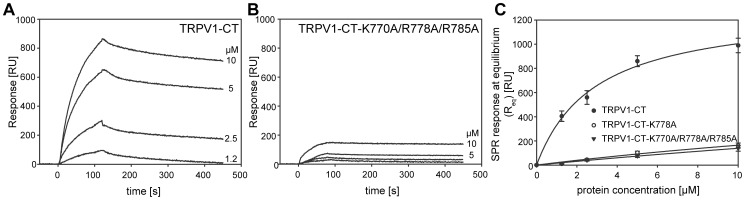
Surface plasmon resonance (SPR) analysis of interactions between TRPV1-CT and PIP2-enriched liposomes. Kinetic binding measurements of TRPV1-CT (A) and the TRPV1-CT-K770A/R778A/R785A triple mutant (B) to the sensor chip coated with PC/PIP2 (80∶20) liposomes. The proteins at indicated concentrations were injected in parallel over the lipid vesicles and the flow rate was maintained at 30 µl/min for both association and dissociation phases of the sensograms. (C) SPR equilibrium binding of the TRPV1-CT, TRPV1-CT-K770A/R778A/R785A, and TRPV1-CT-R778A proteins to the sensor chip coated with PC/PIP2 (80∶20) liposomes. The proteins were injected at 25 µl/min at different concentrations and washed over the lipid surface and Req values were deduced from steady state (equilibrium) SPR response. The solid lines represent binding isotherms determined by nonlinear least-squares analysis of the isotherm using an equation Req = Rmax/1+Kd/P0), where Req stands for SPR response value near -equilibrium, Rmax is the maximum response and P0 is the protein concentration. Values represent the mean ± S.D from four independent experiments.

To provide confirmation of the results from this technique, we used SPR as a different method for the interactions assessment (Fig. 2). Expressed and purified fusion proteins were washed over the liposome-covered chip and equilibrium dissociation constants were estimated (Tab. 1). The dissociation constant for WT TRPV1-CT was 3.0±0.4 µM, which is in a good agreement with the value estimated by steady state fluorescence anisotropy measurement. PIP2 typically interacts with domains containing a cluster of basic amino acid residues. Thus, in order to identify the residues important for TRPV1-CT binding to PIP2, a set of point mutations was performed on the wild-type (WT) DNA, namely single substitutions R778A, R781A, double substitutions K770A/R785A, R771A/R781A, R771A/R778A and triple substitutions K770A/R778A/R785A and K770A/R781A/R785A. The binding of TRPV1-CT point mutants to PIP2 containing liposomes was investigated using SPR measurements (Fig. 2A, B). The most striking effect was the total loss of binding affinity observed for the single mutant R778A, double mutant R771A/R778A and triple mutant K770A/R778A/R785A (Fig. 2C) respectively. Moreover, the K770A/R785A and R771A/R781A mutations decreased the binding affinity, with estimated K_D_ values of 5.8±0.8 µM and 33.5±11.7 µM respectively, compared to the value estimated for the WT TRPV1-CT fusion protein (3.0±0.4 µM as determined by SPR and 3.5±0.9 µM as determined by anisotropy measurement) (Tab. 1).

**Table 1:** Analysis of the effects of charge-neutralizing mutations within the TRPV1-CT distal region (712–838) fusion protein on equilibrium dissociation constants determined by surface plasmon resonance experiments.

Protein	K_D_ [µM]
WT	3.0±0.4
K770A/R781A/R785A	3.6±0.5
K770A/R785A	5.8±0.8
K770A/R778A/R785A	>150
R771A/R781A	33.5±11.7
R771A/R778A	>150
R778A	>150
R781A	9.1±3.8

The presented values are average ± SD from at least 3 independent measurements.

### Effect of Liposome Composition on PIP2 Binding to TRPV1-CT Distal Region

**Figure 3:**
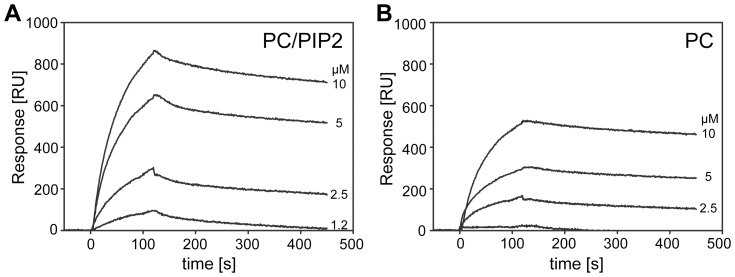
SPR kinetic binding of TRPV1-CT to PIP2-enriched liposomes (A) and to liposomes made from phosphatidyl choline (PC) (B). Both PIP2-enriched (PIP2/PC 80∶20) and PC liposomes were immobilized to the sensor chip at the same density (∼1000 RU), and the TRPV1-CT protein at indicated concentrations was injected in parallel over the lipid vesicles at flow rate of 30 µl/min.

As the TRPV1– CT distal sequence contains several basic amino acids which may interact with anionic lipids, we tested the influence of liposome composition on PIP2 - TRPV1 fusion protein interaction by ELISA. The set of liposomes of different composition was prepared and tested whether there is any dependence of TRPV1 binding on the percent composition of PIP2 in the liposomes. Our results showed the modest selectivity for PIP2 over other used lipids (Figure S1). The control SPR experiments with liposomes containing only phosphatidylcholine(PC) and not containing PIP2 were done and just a weak interaction was detected. (Fig. 3) To check and prove that the binding of TRPV1-CT to PIP2 is (highly) specific and that this protein construct does not bind the phosphatidylcholine molecules, we performed a control experiment using fluorescence anisotropy method with 16∶0-06∶0 NBD PC (1-palmitoyl-2-{6-[(7-nitro-2-1,3-benzoxadiazol-4-yl)amino]hexanoyl}-sn-glycero-3-phosphocholine) and we observed no interaction. (Fig. 1C).

### CaM Binding Site Overlaps with PIP2-Binding Site in TRPV1-CT Distal Region (777–820)

**Figure 4:**
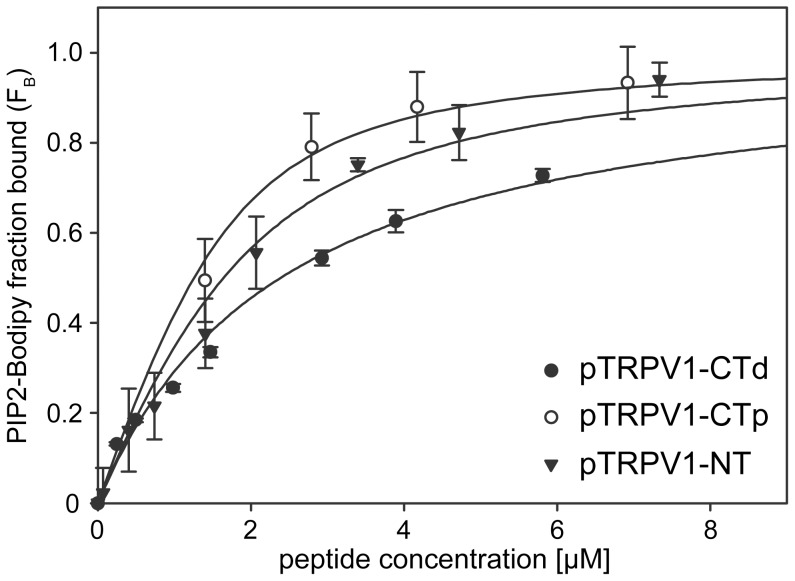
PIP2 recognizes thee independent binding sites within the TRPV1 receptor. Steady-state fluorescence anisotropy measurements of interaction between fluorescently labeled phosphatidyl inositol-4, 5-bisphosphate (PIP2-Bodipy) and synthetic peptides corresponding to cytoplasmic tails either at the N-terminal region F189-V221 of TRPV1 (pTRPV1– NT), C terminal proximal region K688-K718 of TRPV1 (pTRPV1–CTp), or C-terminal distal region L777-S820 of TRPV1 (pTRPV1–CTd), respectively. PIP2-Bodipy (10 nM) was titrated with indicated concentrations of the peptides and the bound fraction (F_B_) of PIP2 Bodipy was calculated according to Equation 1 as described in Material and Methods. The solid lines represent binding isotherms determined by nonlinear least-squares analysis of the isotherm using an Equation 2 as described in Material and Methods. Values represent the mean ± SD from at least three independent experiments.

It has been shown that PIP2 and the CaM-Ca^2+^ complex can bind to unstructured clusters of basic amino acids with high affinity [28], [29]. To investigate whether the binding sites for PIP2 and CaM in TRPV1-CT distal region overlap, we used a synthetic peptide containing only the TRPV1-CT CaM binding site (L777-S820; here denoted as pTRPV1-CTd) [8], [11]. We tested the PIP2 binding to the pTRPV1-CTd peptide using steady-state fluorescence anisotropy measurements. Increasing amounts of TRPV1 - peptides were titrated into a cuvette containing fluorescently labeled PIP2 (Fig. 4). We observed the binding of PIP2 to this peptide and estimated the equilibrium dissociation constant of the pTRPV1-CTd - PIP2 complex to be 1.88+/−0.46 µM (Tab. 2).

**Table 2:** Equilibrium dissociation constants (K_D_) and their standard deviations of synthetic peptides (pTRPV1) of three different regions on the cytoplasmic tails binding to Bodipy® FL C5, C6-PtdIns(4,5)P2 estimated by fluorescence anisotropy measurement.

Peptide	K_D_ [µM]
pTRPV1-CTp	0.328+/−0.06
pTRPV1-CTd	1.88+/−0.46
pTRPV1-NT	1.90+/−0.40

**Figure 5:**
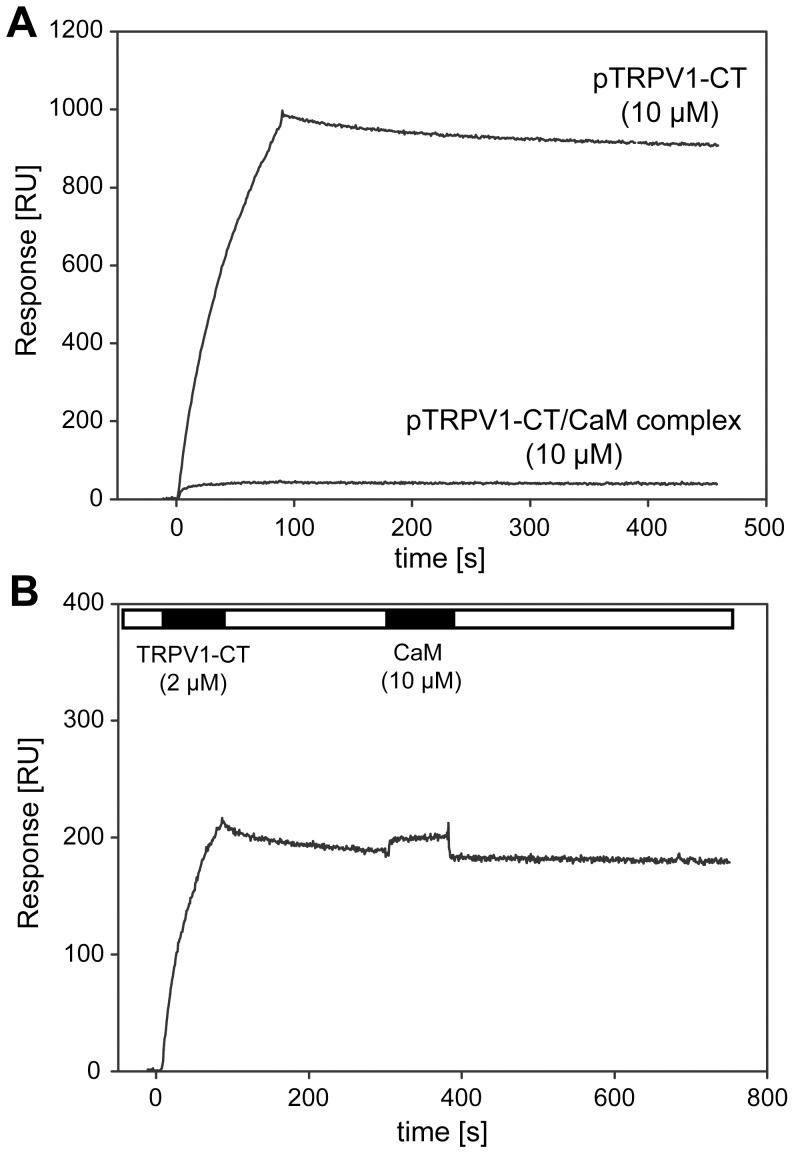
Both PIP2 and calmodulin (CaM) shares the binding site within the C-terminal distal region of TRPV1. (**A**) SPR kinetic binding of TRPV1–CT and the complex of TRPV1–CT with calmodulin (**TRPV1**/CaM complex) to the sensor chip coated with PC/PIP2 (80∶20) liposomes. TRPV1-CT and the TRPV1-CT/CaM complex (both at 10 µM concentration) were injected in parallel over the lipid vesicles and the flow rate was maintained at 30 µl/min for both association and dissociation phase. (**B**) A typical SPR kinetic binding of TRPV1-CT to the PIP2-enriched liposomes followed by independent injection of CaM. TRPV1-CT (2 µM) was injected over the sensor chip coated with PC/PIP2 (80∶20) liposomes, left to dissociate and then calmodulin was injected onto the identical surface at 10 µM concentration. The flow rate was maintained at 30 µl/min during whole experiment. Black and white strips represent association and dissociation phase of the sensogram, respectively.

To confirm that this PIP2 binding site colocalizes precisely with the CaM binding site, we used SPR and tested whether the TRPV1-CT/CaM-Ca^2+^ complex is able to bind PIP2. A protein complex suitable for SPR analysis was prepared and purified (Figure S2). Once liposomes were made and loaded onto the chip, 5 µM TRPV1-CT/CaM-Ca^2+^ complex was washed over the chip. No binding was observed with the protein complex, confirming the colocalization of the PIP2 and CaM binding sites (Fig. 5A). After the regeneration step, the isolated TRPV1-CT fusion protein (712–838) was washed over the chip and binding of approx. 1000 RU was observed. In contrast, further CaM-Ca^2+^ washing over the chip resulted in no change in RU. (Fig. 5B) These data might imply that CaM and PIP2 bind to the same or overlapping binding sites within the TRPV1– CT distal region.

### PIP2 Interacts with TRPV1-CT Proximal Region (688–718)

**Figure 6:**
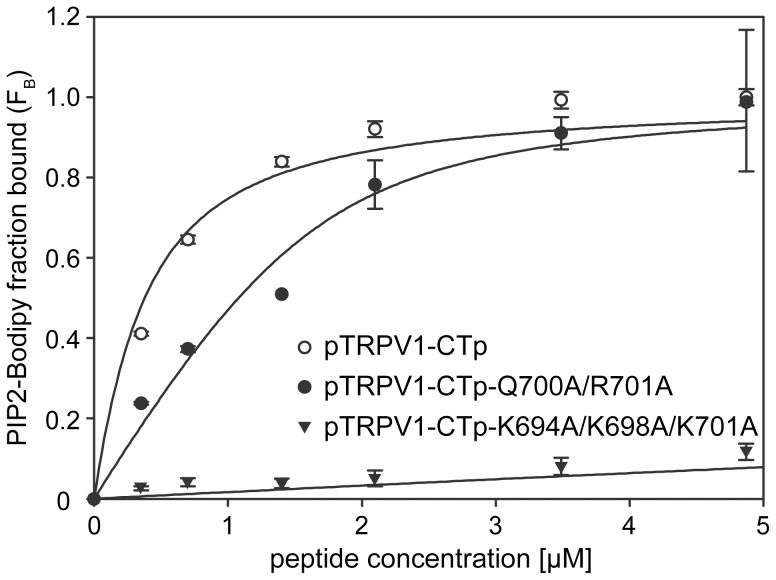
PIP2 binds to the C-terminal proximal region of TRPV1. Steady-state fluorescence anisotropy measurement of interaction between fluorescently labeled phosphatidyl inositol-4, 5-bisphosphate (PIP2-Bodipy) and synthetic peptide corresponding to the cytoplasmic tail at the C terminal proximal region K688-K718 of TRPV1 (pTRPV1–CTp) or its Q700A/R701A (pTRPV1–CTp-Q700A/R701A) and K694A/K698A/K710A (pTRPV1–CTp-K694A/K698A/K710A) mutant variant, respectively. PIP2-Bodipy (10 nM) was titrated with with indicated concentrations of the peptides and the bound fraction (F_B_) of PIP2 Bodipy was calculated according to Equation 1 as described in Material and Methods. The solid lines represent binding isotherms determined by nonlinear least-squares analysis of the isotherm using an Equation 2 as described in Material and Methods. Values represent the mean ± SD from at least three independent experiments.

The TRPV1 – CT proximal region has been proposed to be an important regulatory site [7], moreover it has recently been suggested that PIP2 can interact directly with the proximal region of TRPV1 [22]. To test the PIP2 binding to the proximal part of the TRPV1-CT, we used the fluorescence anisotropy measurement with synthetic peptides (pTRPV1-CTp) corresponding to the wild type (K688-K718) and its point mutants. The mutations were suggested according to the fact that there are eight basic residues in the pTRPV1-CTp sequence that could bind PIP2 electrostatically: K688, K694, K698, R701, K710, K714, R717 and K718. The equilibrium dissociation constant for the wild type peptide was estimated to be 0.814+/−0.171 µM (Table 3). The K694A/K698A/K710A triple mutant seemed to completely lose its binding affinity to PIP2– Bodipy (Fig. 6). Alanine substitutions of the additional candidate residues in the highly conserved QRA region Q700A/R701A significantly attenuated its binding affinity to PIP2-Bodipy (Fig. 6), Indeed these data show that TRPV1-CT proximal region directly binds PIP2 with a high affinity and suggested basic residues play crucial role in the binding.

**Table 3:** Summary of equilibrium dissociation constants (K_D_) and their standard deviations estimated by fluorescence anisotropy measurement of TRPV1-CT proximal region (688–718) wild-type (peptide pTRPV1-CTp) and its mutants.

Peptide	K_D_ [µM]
pTRPV1-CTp	0.328+/−0.06
pTRPV1-CTp K694A/K698A/K710A	>150
pTRPV1-CTp Q700A/R701A	0.814+/−0.17

### PIP2 Binds to the CaM Binding Site Present in TRPV1-NT Ankyrin Repeat Domain (189–221)

Recently, one more CaM binding region of TRPV1 was reported. A TRPV1-NT ankyrin binding domain was suggested to interact with CaM and was described in detail using size exclusion chromatography [15]. The CaM binding regions has been commonly considered as a mutual binding sites for CaM and PIP2. As the PIP2 and CaM interaction regions share key structural features such as interspersed basic and hydrophobic amino acid residues [12], we wondered whether these CaM binding regions of TRPV1 also interact with PIP2. Thus, we designed a peptide that corresponded to this putative binding site – the ankyrin binding domain peptide, pTRPV1-NT (F189-V221). We tested the binding of this peptide to PIP2 by fluorescence anisotropy assay. We observed the binding of PIP2 and estimated the equilibrium dissociation constant of the pTRPV1-NT - PIP2 complex to be 1.9+/−0.4 µM (Tab. 2). The steady-state anisotropy measurement confirmed that the region denoted as CaM interaction site pTRPV1-NT (F189-V221) binds PIP2 with high affinity.

### Molecular Modeling and Ligand Docking

**Figure 7:**
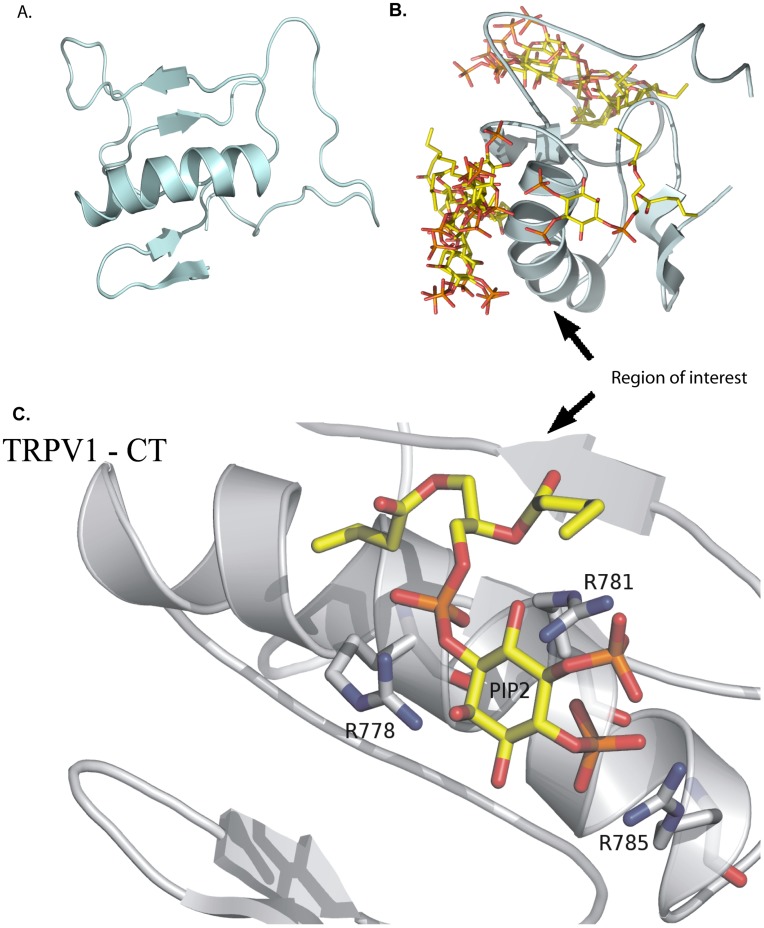
Model of interactions of TPRV1 – CT (V746 - K838) with PIP2. A. Model of TRPV1-CT V746 - K838 generated by homology modeling using Modeller 9v9 software. This model is based on known structure of fragile histidine triad (FHIT) protein from Serin 2 to Aspartate 150 (pdb. accession number: 1FIT). The primary structure of this protein shows a high degree of similarity (44%). **B.** Conformation of the TRPV1 – CT (V746 - K838)/PIP2 complexes after the initial docking using Autodock4 software. Searching was done along the whole surface of the TRPV1 molecule. All suggested TRPV1-CT V746 - K838/PIP2 molecule interaction states are visualized. **C.** Detailed view of the region of interest of TRPV1 – CT (V746 - K838) determined in the initial docking step. Residues hydrogen bonded to PIP2 are highlighted namely R778, R781, R785. All side chains are shown as sticks. The colors representation is following: the backbone of the protein (grey), carbons (yellow), phosphorus (orange) and oxygen (red).

To gain structural insight into these experimentally obtained results, a homology model of TRPV1 C – terminus was created using a restraint-based comparative modeling approach. (Fig. 7A) The stereochemical quality was checked and 94% of the residues are in the most favoured regions of the Ramachandran plot and has an acceptable geometry. The z-score of the protein is −1.1. This value is within the range of scores typically found for proteins of similar size belonging to one of these groups. In order to be able to perform a structural comparison between template structure and homology model molecule the RMSD value was assessesed. To provide the RMSD values of alpha carbons the superimposing of both 3D structures was done using SPDV tool. The RMSD value was calculated to be 1.42 Å.The model of PIP2 molecule which is lacking two aliphatic chains was subsequently docked as PIP2. During the docking procedure the whole molecule of TRPV1 – CT (V746 to K838) was examined and several interaction sites were suggested (Fig. 7B). The 80% of conformations occupy experimentally identified region. Thus in order to gain the detailed insight to the complex forming, the second step of docking procedure was performed. These results provide direct visualization of the interactions between the ligand and nearby atoms in the receptor. In silico binding results are in a good agreement with our experimental work. Our model confirms the crucial interactions between the positively charged residues R778, R781, R785 (Fig. 7C) and PIP2. This is in line with our experimental results, which suggest that R778 plays a pivotal role in this interaction. In our model, the PIP2 molecule also occupies the region previously identified as the binding site for CaM. This region forms an alpha helical structure, as has been predicted before [8].

## Discussion

PIP2 regulates a wide range of cellular functions and the activity of many ion channels including TRP superfamily members [19], [29]. Various domains like PH domains that recognize particular phosphoinositides have been suggested [9], [10], [22], [23] This study identifies two clusters of basic amino acid residues in the carboxy–terminal and one in the amino–terminal cytoplasmic regions that interact with PIP2. It has been known for a long time that PIP2 typically interacts with the Pleckstrin homology domain (PH) [19]. The PH domain contains a cluster of basic amino acid residues and is known to bind phosphoinositides. Similarly the region of interaction between CaM and its cellular targets often possesses a basic helix consisting of approximately 20 amino acids [8], [13], [28], [30]. It has also been shown that the CaM and PIP2 binding regions could overlap in the one of the members of canonical TRP channels subfamily, TRPC6 and moreover that these two ligands could compete for the mutual binding sites. Initially these overlapping binding sites were described in the MARCKS protein family [31].

Our molecular model of the PIP2 interacting with TRPV1 C-terminal distal region suggested that phosphate head groups of PIP2 form polar interactions with positively charged arginines R778, R781, R785. PIP2 thus occupies the CaM binding groove containing R771, R778, R781, R785 as we had described previously [8]. R771A significantly inhibits the CaM binding to the TRPV1-CT fusion protein, nonetheless this construct preserves its binding ability to PIP2. The next single substitution of R778A inhibited the PIP2 binding, significantly increased the dissociation constant but did not prevent CaM binding [8]. These various effects are in a good agreement with the previously described phosphoinositide/CaM interactions with TRPC6. [12] The PH domain typically contains at least several basic residues, which participate in the binding of phosphoinositides, by formation of salt bridges between its positively charged amino acid residues and the phosphate groups of PIP2 [19]. Hence a mutagenesis screening of these residues (K770, R771, R778, R781 and R785) was carried out, combined with SPR measurements. This set of experiments revealed the key role of the R778 and R781 residues in the binding of PIP2. Moreover, further combinations of alanine substitutions revealed that the TRPV1-CT distal region participates in PIP2 binding through a cluster of basic residues: the double and triple substitutions of R771A/R778A, K770A/R778A/R785A avoided PIP2 binding totally and the K770A/R785A and R771A/R781A mutations suppressed this interaction partially.

**Figure 8:**
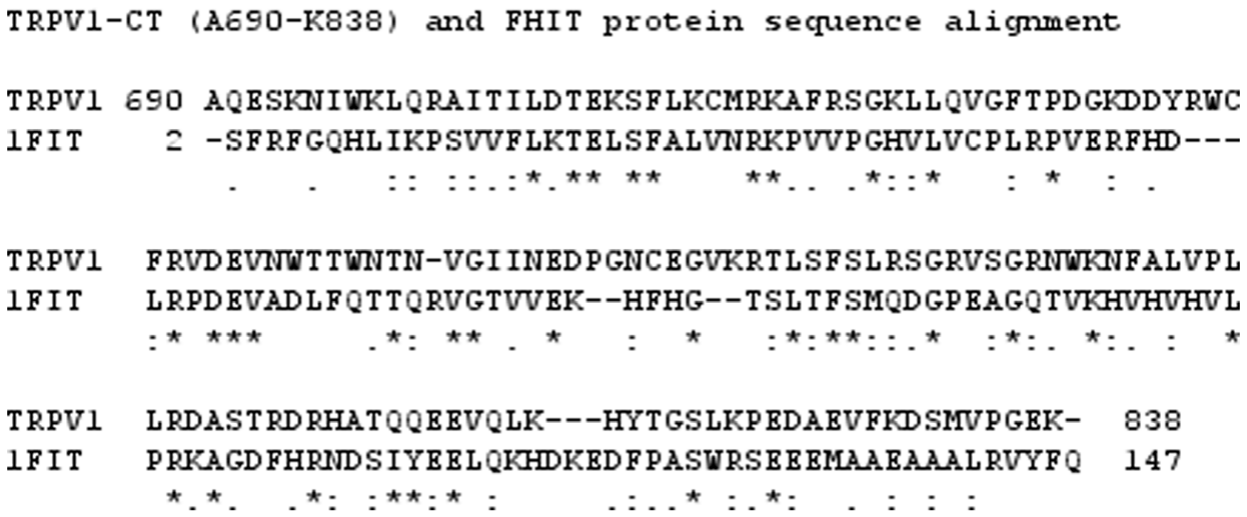
The sequence alignment of the C-terminus of TRPV1 A690 - K838 and the fragile histidine triad protein (FHIT) S2-D150. Identical amino acids are marked with an asterisk. Similar amino acids with the more important groups are indicated with a colon. Dots indicate similar amino acids of the less important groups that are less likely to influence the protein structure.

The TRPV1–CT proximal region K688-K718 motif has been suggested to be an important regulatory site of TRPV1 [7]. We have thus designed the peptides of this region and tested whether its charge neutralization preserves wild-type binding to PIP2. R701 was proposed to be involved in PIP2 dependent activation [7]. Site directed mutation of this arginine reduces binding affinity to PIP2. This is in agreement with previous measurements as the same behavior has been detected in other TRP family members [10]. Moreover we tested other conserved cationic residues within this region that could interact with negatively charged phosphoinositides. The triple substitution at positions K694A/K698A/K710A had the most pronounced effect, completely preventing PIP2 from binding to this region.

It has been previously reported that CaM binds to the ankyrin repeat domain in the N-terminus of TRPV1 [15]. It has also been suggested that PIP2 could be involved in the modulation of TRPV1 functionality via the same region [15]. As the ankyrin repeats sequence shares key structural features with the common PIP2 interaction sites, we tested this region as a possible overlapping binding site for CaM and PIP2. In order to demonstrate the interaction of ankyrin repeats with PIP2, fluorescence anisotropy measurements with synthetized peptide (pTRPV1-NT) were done and the equilibrium dissociation constant was estimated. The resulting K_D_ had an almost identical value to the pTRPV1-CTd peptide K_D,_ representing the C-terminal CaM binding site. Lishko at al. showed that CaM interacts with the ankyrin repeats domains of TRPV1-NT and is involved in channel tachyphylaxis as well as PIP2, but the direct interaction of TRPV1-NT with PIP2 was not confirmed [15]. Here we provide direct evidence that the synthetized peptide (pTRPV1-NT) of the part of the ankyrin domains (F189-V221) binds fluorescently labeled PIP2.

Although a number of studies regarding the physiological significance of the regulation of TRPV1 receptor via PIP2 has been published, its precise molecular mechanism remains unsolved. Here, we have identified multiple PIP2 binding sites within the cytosolic tails of the TRPV1 channel using a combination of biochemical, biophysical and bioinformatical tools. We demonstrated that regions F189-V221 within the N-terminus and K688-K718 and L777-S820 within the C-terminus are involved in PIP2 binding. Interestingly, the F189-V221 and L777-S820 regions overlap with the CaM binding sites suggesting that CaM and PIP2 are competing for the same binding site, which might have implications for regulation of the channel function. The N-terminal F189-V221 occupies the multiple ligands binding site within the ankyrin repeat domain and another binding site lies within the C- terminal CaM binding region. TRPV1 is the first member in the vanilloid subfamily where two regions containing overlapping binding sites for PIP2 and CaM have been identified. Previously, this kind of interaction was shown in the TRPC subfamily [12]. We also revealed a number of amino acid residues within these regions impairing TRPV1-PIP2 interaction. Despite the predicted role of multiple positively charged amino acids in PIP2 binding, we found R778A to have the key role in the interaction. This single mutation leads to a total loss of binding affinity of the distal C- terminal region. Our findings provide a characterization of PIP2 interaction sites and indicate interconnection between the PIP2 and CaM binding to the TRPV1, whose physiological significance may need further investigation.

## Materials and Methods

### Molecular Biology

Part of the sequence of the rat C-tail of TRPV1 (amino acids 712–838) (TRPV1-CT) (NCBI Reference Sequence: NP_114188.1): was subcloned into the pET-32b expression vector (Stratagene). DNA ligation was verified by DNA sequencing. Point mutations of several amino acid residues for Alanine were performed, namely R771A, R778A as well as the double substitutions K770A/R785A, R771A/R781A, R771A/R778A and triple substitutions K770A/R778A/R785A, K770A/R781A/R785A. Mutagenesis PCRs were performed using PfuUltra High-fidelity DNA Polymerase (Stratagene). All mutations were confirmed by DNA sequencing.

### Fusion Protein Expression and Purification

TRPV1-CT was expressed as a fusion protein with thioredoxin tag in Rosetta *Escherichia coli* cells. Protein expression was induced by isopropyl-1-thio-D-galactopyranoside (Roth) for 12 h at 20°C. Cells were disrupted by sonication and the protein waspurified using Chelating Sepharose Fast Flow (Amersham Pharmacia Biotech) according to the standard manufacturer’s protocol. Protein samples were concentrated using spin columns (Millipore). The subsequent purification step was a gel permeation chromatography on a Superdex 200 column (Amersham Pharmacia Biotech). Protein concentration was assessed by the measuring of absorption at 280 nm. The purity was verified by using 15% SDS- polyacrylamide gel electrophoresis (PAGE).

### Calmodulin (CaM)/TRPV1 – CT Complex Preparation

Mouse CaM was expressed from the pET3a vector in BL21 *Escherichia coli* cells. Protein expression and purification were done according to the protocol described in our previous study [32]. Binding the recombinant TRPV1 fragment (sequence 712–838) to CaM was done in the presence of CaCl_2_. The protein mixture, with a 1∶1 molar ratio of TRPV1 to CaM was incubated for 1 h at room temperature (RT). An additional purification step was subsequently done to separate the unbound fraction, gel permeation chromatography in a Superdex 75 column (Amersham Pharmacia Biotech).

### Steady State Fluorescence Anisotropy Binding Assay

The fusion protein of TRPV1-CT (712–838) was prepared, the series of synthetic peptides (pTRPV1) of three different regions of the cytoplasmic tails of the TRPV1 channel (NCBI Reference Sequence: NP_114188.1) and its mutations were obtained from GenScript USA Incorporated, New Jersey, namely: TRPV1 – CT proximal region K688-K718 (pTRPV1–CTp) (KIAQESKNIWKLQRAITILDTEKSFLKCMRK), TRPV1 – CT proximal region K688-K718 possessing mutations of K694A/K698A/K710A (KIAQES**A**NIW**A**LQRAITILDTE**A**SFLKCMRK), TRPV1 – CT proximal region K688-K718 possessing mutations of Q700A/R701A (KIAQESKNIWKL**AA**AITILDTEKSFLKCMRK), TRPV1 – CT distal region L777-S820 (pTRPV1 – CTd) (LRSGRVSGRNWKNFALVPLLRTDASTRDRHATQQEEVQLKHYTGS), TRPV1 – NT region F189-V221 (pTRPV1 – NT) (FVNASYTDSYYKGQTALHIAIERRNMTLVTLLV). Peptides were dissolved in 50 mM Tris-HCl (pH 7.5), 100 mM NaCl buffer and were used for measurements of fluorescence anisotropy. Steady-state fluorescence anisotropy measurements were performed in an ISS Photon Counting Steady-State Spectrofluorimeter (ISS PC1TM) at room temperature with a PIP2-Bodipy® FL C5, C6-PtdIns(4,5)P2 molecular probe (Invitrogen, cat. n. B22627, Figure S3A), hereafter denoted as PIP2-Bodipy and the control experiment was done with 16∶0-06∶0 NBD PC 1-palmitoyl-2-{6-[(7-nitro-2-1,3-benzoxadiazol-4-yl)amino]hexanoyl}-sn-glycero-3-phosphocholine in chloroform (NBD-PC), (Avanti Polar Lipids, Inc. Cat. n. 810130C, Figure S3B) The PIP2-Bodipy probe was diluted in deionized H_2_O. The final concentration of the fluorescently labeled probe in the measuring buffer in the cuvette was 10 nM and increasing amounts of peptides and fusion protein TRPV1 respectively were titrated into the cuvette. PIP2-Bodipy excitation and emission wavelengths were set to 500 nm and 512 nm and for NBD – PC 460 nm and 534 nm respectively. At each peptide concentration, steady-state fluorescence anisotropy was recorded. The fraction of TRPV1s regions or their mutants bound to fluorescent probe was determined from the anisotropy changes using Eq. (1) [33], where F_B_ is the fraction bound, r_min_ and r_max_ are the anisotropies of the free and bound PIP2-Bodipy with TRPV1-CT, pTRPV1 regions or its mutants, r_obs_ is the observed anisotropy, and Q is the ratio of the intensities of the free and bound protein (f_max_/f_min_). All experiments were carried out in at least triplicate.
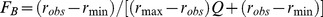
(1)


### Dissociation Constant Assessment

The F_B_ was plotted against the TRPV1 peptides and fusion proteins concentration respectively and fitted using Eq. (2) [33] to determine the equilibrium dissociation constant (K_D_). Non-linear data fitting was performed using the package SigmaPlot 2000 (6.1) SPSS Inc. P_1_ is the concentration of PIP2-Bodipy and P_2_ is the concentration of TRPV1 fusion protein or TRPPV1 synthetic peptides.
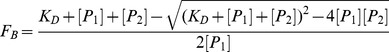
(2)


### Liposome Preparation

The lipids L-α-phosphatidylinositol-4,5-bisphosphate (PIP2), 1,2-dimyristoyl-sn-glycero-3- Phosphatidylserine (PS), 1,2-dimyristoyl-sn-glycero-3-phosphocholine (PC) and 1,2-dimyristoyl-sn-glycero-3- Phosphatidylethanolamine (PE) were obtained from Avanti Polar Lipis, Inc. A stock solution of each lipid was prepared in chloroform, except for PIP2 stock solution, which was prepared in a chloroform:methanol (2∶1) mixture. Liposomes of the following compositions were prepared: PC, PC/PIP2 97/3%, PC/PIP2 90/10%, PC/PIP2/PS 70/10/20%, PC/PIP2/PS/PE 40/10/20/30%, PC/PIP2/PE 70/10/20%, PC/PIP2/PE 77/3/20%, PC/PE/PS 50/30/20% by mixing appropriate volumes of the stock solutions. After being dried under an N_2_ stream, lipid films were hydrated with 1× HBSS buffer containing 8 µM oligonucleotide 5′-TATTTCTGATGTCCACCCCC-3′, modified at the 3′ end with cholesterol (Generi-Biotech, Hradec Kralove, Czech Republic)- followed by extrusion 20 times through a polycarbonate membrane with a 100-nm pore diameter (Avestin Europe, Mannheim, Germany). Finally 8 µM complementary oligonucleotide Biotin conjugate 5′-TGGACATCAGAAATACCCCC-3′ (Generi-Biotech, Hradec Kralove, Czech Republic) was added to the liposome mixture. A 20-min incubation was followed by centrifugation and washing steps to remove the unbound oligonucleotide-Biotin conjugate.

### Elisa

96-well micro titer plates were used. Each well was coated with 100 µl of (20 µg/ml) C-tail of TRPV1 (amino acids 712–838) in coating buffer (1×HBSS buffer) or standard (PIP Grip -Avanti Phospholipis) and incubated for 8 h at 4°C. The coating solution was shaken out of the wells and the wells were blocked with 100 µl 1% BSA per well and incubated O/N at 4°C. The next day all eight types of liposomes were added and 2-fold dilutions up to 1/64× were performed. Liposomes were incubated for 1 hour at room temperature. The unbound liposomes were disposed off and the wells were washed with 1× HBSS buffer. Streptavidin/Horseradish peroxidase conjugate at 1∶3000 dilution was subsequently added and incubated at room temperature for 1 hour followed by the next 1× HBSS washing step and the addition of substrate solution (H_2_O_2_, o-phenylenediamine (OPD), citrate buffer). The substrate was freshly prepared by adding 40 mg/100 ml of OPD in citrate buffer, and 2 µl of 30% H_2_O_2_ was added to each ml of OPD solution. Thereafter, the plates were further incubated at room temperature in the dark for exactly 15 minutes. The reaction was stopped by adding 50 µl of H_2_SO_4_ into each well. Absorbance was read at 490 nm using a micro plate reader.

### Surface Plasmon Resonance

All SPR measurements were performed at 25°C using a liposome-coated NLC chip in aProteOn XPR36 Protein Interaction Array System (Bio-rad, Hercules, CA, USA). Liposomes (100 nm in diameter) were made from 1,2-dimyristoyl-sn-glycero-3-phosphocholine (PC) and L-α-phosphatidylinositol-4,5-bisphosphate (PIP2) (Avanti Lipids, Alabaster, AL, USA) at a molar ratio of 20∶1 (PC/PIP2). Lipid solution (10 mg/ml) in HBSS was mixed with 8 µM oligonucleotide 5′-TATTTCTGATGTCCACCCCC-3′, modified at the 3′ end with cholesterol (Generi-Biotech, Hradec Kralove, Czech Republic), and the mixture was extruded using a LiposoFast Basic apparatus (Avestin Europe, Mannheim, Germany) with a polycarbonate membrane with a 100-nm pore diameter (Avestin Europe, Mannheim, Germany). The lipid/DNA ratio was 1200∶1. The vesicles were then incubated with 8 µM anti-sense oligonucleotide 5′-TGGACATCAGAAATACCCCC-3′, modified at the 5′ end with biotin (Generi-Biotech, Hradec Kralove, Czech Republic), and washed by centrifugation (50,000 g) for 30 min at 4°C. The vesicles were diluted to a final concentration of 1 mg/ml in HBSS and immobilized on the streptavidin-coated NLC chip (Bio-rad, Hercules, CA, USA) surface at a flow rate of 25 µl/min for 10 min to give ∼800 −1500 resonance units (RU). All SPR measurements were carried out in HBSS at a flow rate of 30 µl/min for both the association and dissociation phase of the sensograms. For determining equilibrium binding, the association phases of five different concentrations of each protein were brought to near-equilibrium values (Req). Surfaces were typically regenerated with 50 µl of 1 M NaCl and 20 mM glycin buffer, pH 3.5. The sensograms were corrected for sensor background by interspot referencing (the sites within the 6×6 array which are not exposed to ligand immobilization but are exposed to analyte flow), and double referenced by subtraction of the analyte using a “blank” injection. Assuming a Langmuir-type binding between the protein (P) and protein binding sites (S) on vesicles (i.e. P + S ↔ PS), Req values were then plotted versus protein concentration (P0), and the K_D_ value was determined by nonlinear least-squares analysis of the binding isotherm using the equation Req  =  Rmax/(1+K_D_/P0).

### Molecular Modeling and Ligand Docking

A comparative homology model of the three-dimensional structure of the cytoplasmic region (from V746 to K838) of the rat C-terminus TRPV1 was generated using Modeller 9.9 software. [34] As there is no known solved structure of any of the C- terminal regions of the the TRPV channel subfamily, we used the crystal structure of fragile histidine triad protein (FHIT, PDB code 1FIT) as a template [35], this template structure has been used for modeling of the C – terminal region in previous works. Sequence similarity between the target (TRPV1 - CT) and the template (FHIT) is 44%, here we use the truncated tail of TRPV1- CT that possesses a 44% sequence similarity as well. The sequences were aligned with CLUSTALX 2.0.10 [36] (Fig. 8). The energy minimization of all models was performed using a Swiss-PdbViewer with the GROMOS96 force field [37] and checked with ProSA-web [38] for recognizing errors in the 3D protein structure. The docking of this ligand (PDB code 3SPI) [39] to the C-terminus of TRPV1 was performed to obtain a population of possible conformations and orientations for the ligand at the binding site using Autodock 4. The Lamarckian genetic algorithm method was employed for docking a flexible ligand and rigid protein. In order to obtain receptor–ligand complexes, a two step strategy was used. In the starting point for ligand docking less restrictive conditions were set up, the grid box was centered to the macromolecule allowing ligand to explore the whole macromolecule. The cluster with the highest population of suggested ligand-receptor conformation was then seleted. This region served as a starting point for the second step of the docking procedure.To show the interactions more precisely a grid box with 40, 40 and 40 points in the x, y, and z directions was built to cover the entire suggested binding site and accommodate ligands to move freely with a grid spacing of 0.375 Å. The default settings were used for all other parameters. The best conformation with the lowest docked energy was chosen.

## Supporting Information

Figure S1 A. Schema of Elisa. TRPV1 fusion protein was non-specifically immobilized via adsorption to the surface of a microtiter plate. After the imobilization, the liposomes were added, forming a complex with the fusion protein. Each liposome had incorporated the cholesterol/oligonucleotide conjugate, which is complementary to the biotinylated oligonucleotide. The plate was developed by adding an enzymatic substrate (streptavidin/horse radish peroxidase) to produce a visible signal. B. The graph compares liposomes of different composition (PC80%PIP20% -triangles, PC97%PIP3% - white circles, PC – black circles) and the corresponding binding of the TRPV1-CT fusion protein.
(DOCX)

Figure S2 **TRPV1-CT/CaM complex formation.** Chromatogram from size exclusion chromatography including Coomassie-stained 15% SDS-PAGE of fractions 7–9.
(DOCX)

Figure S3 A. Molecular structure of PIP2-Bodipy® FL C5, C6-PtdIns(4,5)P2 molecular probe (Invitrogen, cat. n. B22627) B. Molecular structure of 16∶0-06∶0 NBD PC 1-palmitoyl-2-{6-[(7-nitro-2-1,3-benzoxadiazol-4-yl)amino]hexanoyl}-sn-glycero-3-phosphocholine in chloroform (NBD-PC), (Avanti Polar Lipids, Inc. Cat. n. 810130C).
(DOCX)
