# Supplementary figures and images for: Integrative Binding Sites within Intracellular Termini of TRPV1 Receptor
Source: PLoS One. 2012 Oct 31;7(10):e48437. doi: 10.1371/journal.pone.0048437 (PMC3485206; doi:10.1371/journal.pone.0048437)

Figure S1


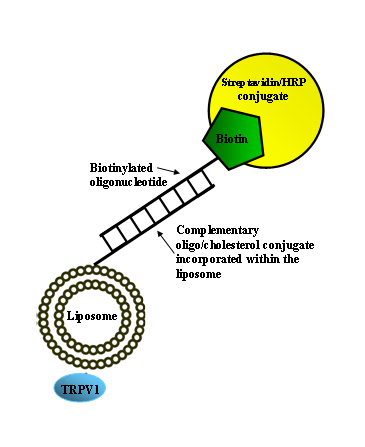


**A.**


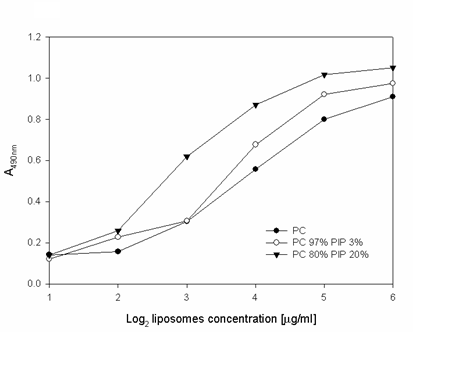


**B**.

Supplement: Figure S1 — A. Schema of Elisa. TRPV1 fusion protein was non-specifically immobilized via adsorption to the surface of a microtiter plate. After the imobilization, the liposomes were added, forming a complex with the fusion protein. Each liposome had incorporated the cholesterol/oligonucleotide conjugate, which is complementary to the biotinylated oligonucleotide. The plate was developed by adding an enzymatic substrate (streptavidin/horse radish peroxidase) to produce a visible signal. B. The graph compares liposomes of different composition (PC80%PIP20% -triangles, PC97%PIP3% - white circles, PC – black circles) and the corresponding binding of the TRPV1-CT fusion protein. (DOCX) [file pone.0048437.s001.docx]

**Figure S2**


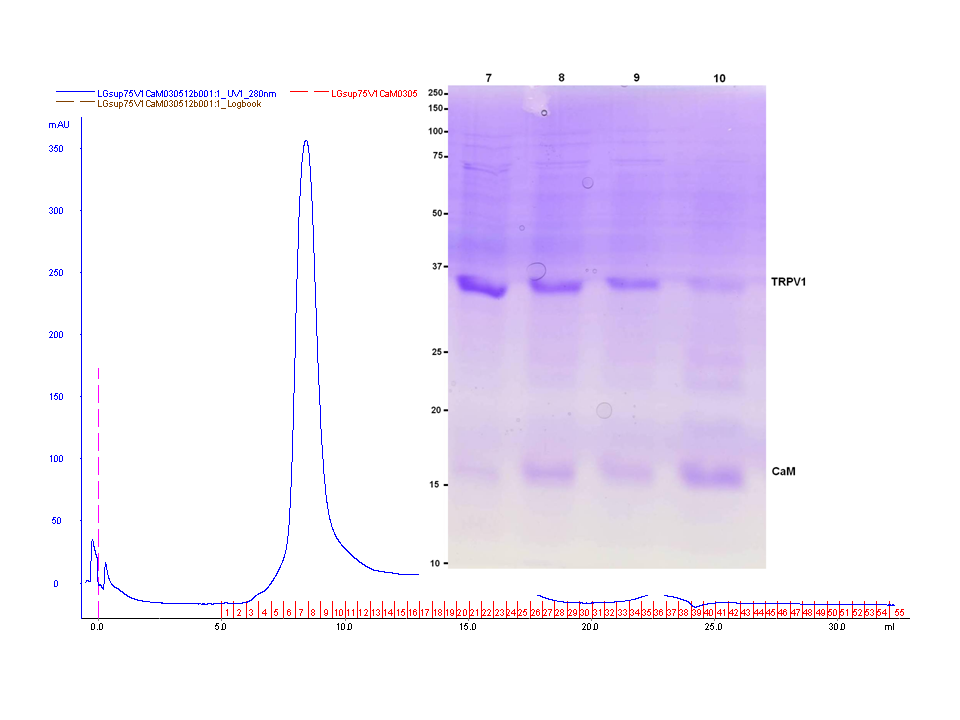

Supplement: Figure S2 — TRPV1-CT/CaM complex formation. Chromatogram from size exclusion chromatography including Coomassie-stained 15% SDS-PAGE of fractions 7–9. (DOCX) [file pone.0048437.s002.docx]

Figure S3

A.


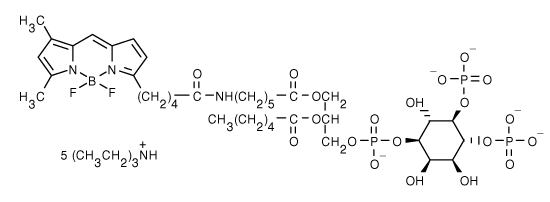


B.


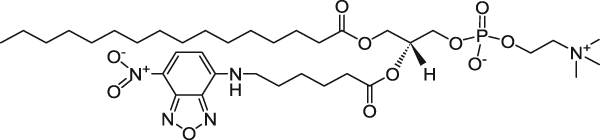

Supplement: Figure S3 — A. Molecular structure of PIP2-Bodipy® FL C5, C6-PtdIns(4,5)P2 molecular probe (Invitrogen, cat. n. B22627) B. Molecular structure of 16∶0-06∶0 NBD PC 1-palmitoyl-2-{6-[(7-nitro-2-1,3-benzoxadiazol-4-yl)amino]hexanoyl}-sn-glycero-3-phosphocholine in chloroform (NBD-PC), (Avanti Polar Lipids, Inc. Cat. n. 810130C). (DOCX) [file pone.0048437.s003.docx]
